# The Widely Conserved *ebo* Cluster Is Involved in Precursor Transport to the Periplasm during Scytonemin Synthesis in *Nostoc punctiforme*

**DOI:** 10.1128/mBio.02266-18

**Published:** 2018-11-27

**Authors:** Kevin Klicki, Daniela Ferreira, Demetra Hamill, Blake Dirks, Natalie Mitchell, Ferran Garcia-Pichel

**Affiliations:** a School of Life Sciences, Arizona State University, Tempe, Arizona, USA; b Center for Fundamental and Applied Microbiomics, Biodesign Institute, Arizona State University, Tempe, Arizona, USA; University of Washington; Oklahoma State University; University of Oxford; Arizona State University

**Keywords:** alkaloids, cyanobacteria, *ebo* genes, excretion, lipid carriers, membrane transport, periplasm, scytonemin, secondary metabolism, sunscreens

## Abstract

Scytonemin is a dimeric indole-phenol sunscreen synthesized by some cyanobacteria under conditions of exposure to UVA radiation. While its biosynthetic pathway has been elucidated only partially, comparative genomics reveals that the scytonemin operon often contains a cluster of five highly conserved genes (*ebo* cluster) of unknown function that is widespread and conserved among several bacterial and algal phyla. We sought to elucidate the function of the *ebo* cluster in the cyanobacterium Nostoc punctiforme by constructing and analyzing in-frame deletion mutants (one for each *ebo* gene and one for the entire cluster). Under conditions of UVA induction, all *ebo* mutants were scytoneminless, and all accumulated a single compound, the scytonemin monomer, clearly implicating all *ebo* genes in scytonemin production. We showed that the scytonemin monomer also accumulated in an induced deletion mutant of *scyE*, a non-*ebo* scytonemin gene whose product is demonstrably targeted to the periplasm. Confocal autofluorescence microscopy revealed that the accumulation was confined to the cytoplasm in all *ebo* mutants but that that was not the case in the *scyE* deletion, with an intact *ebo* cluster, where the scytonemin monomer was also excreted to the periplasm. The results implicate the *ebo* cluster in the export of the scytonemin monomer to the periplasm for final oxidative dimerization by ScyE. By extension, the *ebo* gene cluster may play similar roles in metabolite translocation across many bacterial phyla. We discuss potential mechanisms for such a role on the basis of structural and phylogenetic considerations of the *ebo* proteins.

## Abstract (precis)

Elucidating the biochemical and genetic basis of scytonemin constitutes an interesting challenge because of its unique structure and the unusual fact that it is partially synthesized in the periplasmic space. Our work points to the *ebo* gene cluster, associated with the scytonemin operon of cyanobacteria, as being responsible for the excretion of scytonemin intermediates from the cytoplasm into the periplasm during biosynthesis. Few conserved systems have been described that facilitate the membrane translocation of small molecules. Because the *ebo* cluster is well conserved among a large diversity of bacteria and algae and yet insights into its potential function are lacking, our findings suggest that translocation of small molecules across the plasma membrane may be its generic role across microbes.

## INTRODUCTION

The cytoplasm is an ideal environment for the synthesis of secondary metabolites, being highly regulated and rich in energetic compounds, enzymes, and cofactors. Often, however, products synthesized there must function in the periplasm or outside the cell, necessitating transmembrane systems to facilitate their transport through the cytoplasmic membrane. Among the secondary metabolites that are synthesized in the cytoplasm but later excreted are some sunscreen compounds produced by cyanobacteria to cope with excess deleterious radiation. Some species growing on exposed surfaces produce UV-absorbing sunscreens, e.g., mycosporine-like amino acids and scytonemin, which intercept UV radiation and prevent damage to cellular machinery (1); the latter may also exhibit anti-inflammatory activity (2). Scytonemin, found exclusively among cyanobacteria, is a brownish-yellow, lipid-soluble pigment that is excreted and accumulated in the extracellular matrix in response to UVA radiation (315 to 400 nm) (3–5). Structurally unique among natural products, it is a homodimeric indole-alkaloid, with a molecular mass of 544 g mol^−1^ in its oxidized, active form, and is composed of two heterocyclic units symmetrically connected through a carbon-carbon bond (6). The complex ring structure allows strong absorption in the UVA-violet-blue range (325 to 425 nm), with a maximum level at 384 nm in acetone and around 370 nm *in vivo* (3, 6).

**FIG 1:**
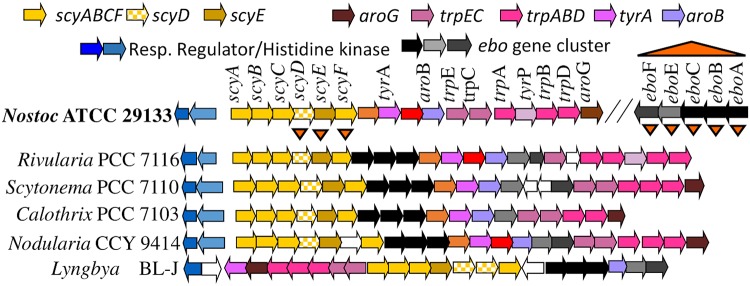
Genomic organization of the scytonemin operon in N. punctiforme and other cyanobacteria, including the *ebo* genes of unknown function found within the *scy* operon of most cyanobacteria (but distally in N. punctiforme*).* Triangles indicate the genes whose deletion mutants were examined in this study.

**FIG 2:**
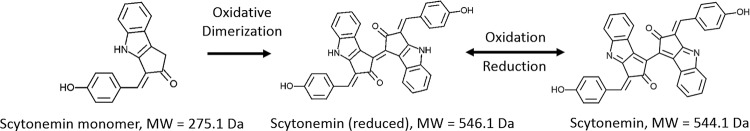
Structures of the scytonemin monomer and scytonemin. The likely final step in scytonemin biosynthesis involves oxidative dimerization of the scytonemin monomer to yield reduced scytonemin, which undergoes facile auto-oxidation to scytonemin proper. MW, molecular weight.

A genomic region comprising 18 contiguous open reading frames (ORFs) (Nostoc punctiforme R1276 [Npun_R1276] to Npun_R1259; Fig. 1) is responsible for scytonemin biosynthesis in N. punctiforme ATCC 29133 (PCC 73102), their transcription being induced by UVA (7–9). Six consecutive genes (ORFs Npun_R1276 to Npun_R1271; named *scyABCDEF* [10]) form the core biosynthetic locus. *In vitro* studies confirmed that ScyA, ScyB, and ScyC carry out the early stages of the scytonemin assembly: ScyB first catalyzes the oxidative deamination of l-tryptophan to yield indole-3-pyruvic acid, while ScyA mediates the acyloin coupling of indole-3 pyruvic acid and *p*-hydroxyphenylpyruvic acid, producing a labile β-ketoacid compound (11). Subsequently, ScyC catalyzes the cyclization and decarboxylation of the previous compound to form a ketone (12), which is one (auto)oxidation state away from what we call the scytonemin monomer (Fig. 2). The precursors are supplied by a set of redundant orthologues coding for enzymes in the aromatic amino acid biosynthetic and shikimic acid pathways (7, 8, 10).

A two-component regulatory system controls the expression of the entire operon (13). While it would logically follow that the rest of the core genes (*scyDEF*) catalyze the final oxidative dimerization of the scytonemin synthesis, the following two lines of evidence indicate that this is not the case: (i) of the three, only *scyE* is essential for scytonemin synthesis (14); (ii) expression of the *scyA–E* locus as well as the entire 18-gene cluster in Escherichia coli was insufficient to attain heterologous scytonemin production (15). Comparative genomics revealed an additional group of five highly conserved genes (*ebo* genes; see below) of unknown function within the *scy* operon of many cyanobacteria. In the genome of N. punctiforme ATCC 29133, however, these are found at a distal locus (Fig. 1) but are also upregulated with the scytonemin synthesis operon under conditions of UVA exposure (1, 9, 10). For these reasons, it was suggested that the *ebo* genes may be involved in the synthesis of scytonemin, perhaps being responsible for the later biosynthetic steps (14).

Intriguingly, this five-gene cluster is conserved in synteny and sequence homology among many bacteria across several phyla as well as in the plastid genomes of some eustigmatophyte algae and hence was named the “eustigmatophyte/bacterial operon,” or *ebo* (16). In-depth bioinformatic analysis, however, did not reveal a clear potential function for the *ebo* genes (16). Their widespread presence in an array of bacterial and plastid genomes (16), the overwhelming majority of which do not produce scytonemin, weakens the hypothesis that they have a dedicated role in scytonemin biosynthesis.

We sought to elucidate the function of the *ebo* gene cluster in N. punctiforme by constructing in-frame deletion mutants in relevant open reading frames and investigating the resulting phenotypes.

## RESULTS

**FIG 3:**
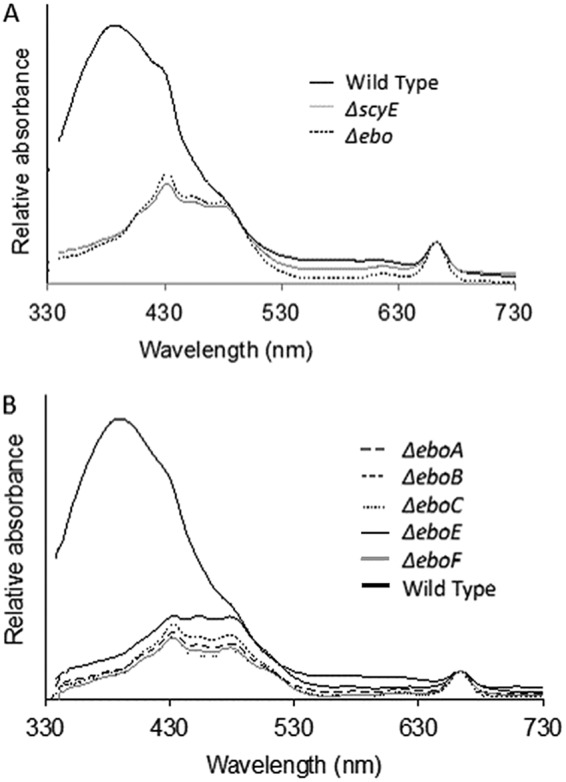
(A) Absorbance spectra of acetone cell extracts from wild-type (solid black), *scyE* mutant (solid gray), and *ebo* mutant (dotted black) strains after UVA induction of the scytonemin operon. The wild-type strain produced scytonemin, as indicated by a large absorbance maximum at 384 nm. (B) Absorbance spectra of acetone cell extracts of individual *ebo* gene deletion mutants after UVA induction of the scytonemin operon, all displaying a scytoneminless phenotype.

To determine whether the product of any gene within the *ebo* cluster (here defined as ORFs from Npun_F5232 to Npun_F5236) was involved in the production of scytonemin, we deleted the entire cluster in N. punctiforme (Fig. 1). The mutant strain, Δ*ebo*, was then tested against the wild-type (WT) strain and a previously obtained scytoneminless mutant, *ΔscyE*, as controls for its ability to produce scytonemin. The Δ*ebo* strain presented a clear scytoneminless phenotype, like that of *ΔscyE*, and only the WT produced scytonemin under inductive conditions (Fig. 3A). Subsequently, to determine which gene products in the *ebo* cluster were responsible for the Δ*ebo* scytoneminless phenotype, and to assess their specific roles, we constructed the following five in-frame deletion mutants: strains Δ*eboA*, Δ*eboB*, Δ*eboC*, Δ*eboE*, and Δ*eboF* (NpunF5232, NpunF5233, NpunF5234, NpunF5235, and NpunF5236, respectively; Fig. 1), in which the rest of the operon was conserved in its proper reading frame (see Table S1 in the supplemental material for details on construction). Recombinant plasmids were sequenced to ensure that no other mutations were created during construction. Chromosome segregation of the deletion mutants was confirmed by PCR using different combinations of primers (Table S1), as previously described (14). We found no polar transcriptional effects by reverse transcriptase PCR (RT-PCR) targeting transcripts of the *ebo* gene downstream of each mutation (see Fig. S7 in the supplemental material), although the steady-state quantities of transcripts were not assessed. All mutant strains were tested for scytonemin production as described for the Δ*ebo* strain, and each of the five in-frame deletion mutants was scytoneminless, indicating that all five *ebo* genes are essential for scytonemin production (Fig. 3B). In addition to lacking scytonemin, the *ebo* deletion mutants also showed enhanced susceptibility to UVA damage under inductive conditions; within the 5-day induction period, all *ebo* mutants exhibited chlorotic phenotypes, while the wild-type strain remained unaffected. This was not necessarily a result of lack of sunscreen, given that other scytoneminless mutants do not exhibit increased sensitivity to UVA (7).

10.1128/mBio.02266-18.9 TABLE S1 Primers used in this work for mutant construction and validation. Download Table S1, PDF file, 0.6 MB. Copyright © 2018 Klicki et al. 2018 Klicki et al. This content is distributed under the terms of the Creative Commons Attribution 4.0 International license.

**FIG 4:**
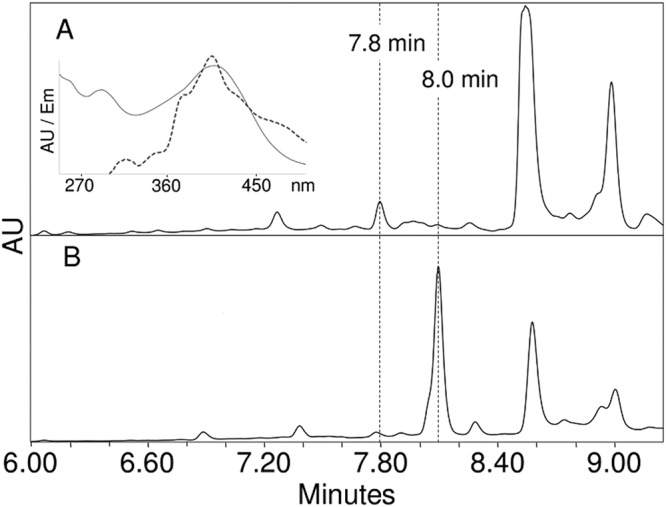
Separation and characterization of a compound accumulated after UVA induction by the Δ*eboC* strain. (A) HPLC chromatogram of acetone extract showing production of a novel compound eluting at 7.8 min and the absence of a scytonemin peak at 8 min. This pattern was found in all *ebo* mutants and in the Δ*scyE* strain (see Fig. S1 in the supplemental material), and none of the strains produced the compound without an induction of the syctonemin operon (see Fig. S1). The inlay shows the UV-visible light (UV-Vis) absorbance spectrum (solid line) and the fluorescence emission (Em) spectrum (dotted line) of the newly accumulated compound after collection from HPLC eluent. AU, absorbance units. (B) Chromatogram of wild-type extract after UVA induction, indicating the presence of scytonemin at 8 min and the absence of the 7.8-min peak. λ = 407 nm.

No compounds unique to the *ebo* mutants were found to accumulate in the aqueous extract preparations in any of the mutants by high-performance liquid chromatography (HPLC) analysis. By contrast, acetone extracts of all *ebo* mutants (single-gene and cluster mutants) contained a single compound that accumulated consistently when induced by UVA exposure but that was not present in the wild-type cell extracts. It exhibited a retention time of 7.8 min and a characteristic absorbance spectrum with a visible maximum at 407 nm (Fig. 4A). This compound was not detected in any of the *ebo* mutants when noninduced cells were extracted (Fig. S1). Upon isolation and collection, it was determined to have a molecular mass of 275 Da (Fig. S2 [mass fragment of 274 Da due to deprotonation]). The retention time, absorbance spectra, and mass are consistent with those of the compound produced by the expression of *scyA–C* in Escherichia coli (15), the structure of which has been fully resolved by nuclear magnetic resonance {(3Z)-3-[(4-hydroxyphenyl)methylidene]-1H,2H,3H,4H-cyclopenta[b]indol-2-one; scytonemin monomer in Fig. 2}. In order to confirm the identity of the accumulated compound, we obtained an authentic standard for HPLC by constructing an E. coli strain containing *scyA–C*, as described in Materials and Methods, and isolating the main compound produced. HPLC coelution of the standard and of each of the compounds collected from *ebo* mutants produced single peaks in all cases, thus confirming their identity. Having determined that the scytonemin monomer accumulated in all of the *ebo* mutants, we also reanalyzed mutants *ΔscyD*, *ΔscyE*, and *ΔscyF*. We could confirm that neither the *ΔscyD* mutant nor the *ΔscyF* mutant (both with a scytonemin-positive phenotype) accumulated the monomer, but we could clearly detect and identify it in the *ΔscyE* mutant under conditions of UV induction (but not in noninduced cells; Fig. S1); this had not been detected in previous studies (14). All *ebo* mutants and the *ΔscyE* mutant were biochemically identical with respect to a lack of scytonemin production and the accumulation of the scytonemin monomer. The presence of the scytonemin monomer in *ΔscyE* cells indicates that absence of *ebo* genes is not an absolute requirement for the production of the scytonemin monomer.

10.1128/mBio.02266-18.2 FIG S1 (A) HPLC chromatograms of *ebo* and *scyE* deletion mutant acetone extracts, after UVA induction, exhibiting a novel compound at 7.8 min. (B) HPLC chromatograms of acetone extracts from uninduced cells of *ebo* and *scyE* deletion mutants. Download FIG S1, PDF file, 0.2 MB. Copyright © 2018 Klicki et al. 2018 Klicki et al. This content is distributed under the terms of the Creative Commons Attribution 4.0 International license.

10.1128/mBio.02266-18.3 FIG S2 (Top) Electrospray ionization time of flight mass spectroscopy analysis in negative-ion mode of blank HPLC solvent. A *ΔeboC* compound eluted at 7.8 min in HPLC after eluent collection (middle). (Bottom) Prediction of mass spectral signal, given the chemical formula of the scytonemin monomer negative-ion fragment. Download FIG S2, PDF file, 0.03 MB. Copyright © 2018 Klicki et al. 2018 Klicki et al. This content is distributed under the terms of the Creative Commons Attribution 4.0 International license.

**FIG 5:**
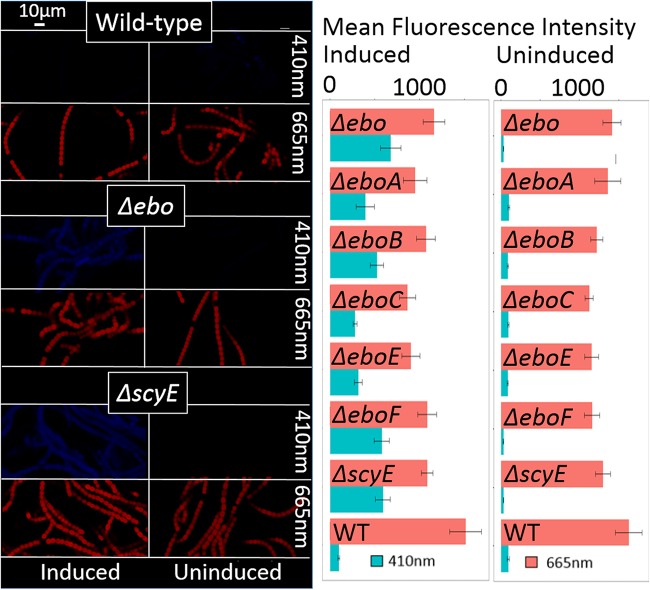
Confocal fluorescence imaging and quantification of the scytonemin monomer accumulation *in vivo*. (Left) Fluorescence images of the wild-type (top), Δ*ebo* (middle), and Δ*scyE* (bottom) strains with emission at 410 nm (to visualize the scytonemin monomer) and 665 nm (to visualize photopigments in the cytoplasm), under conditions of induction and without induction by UVA. (Right) Fluorescence intensity quantification within cells of the wild-type and mutant strains at 410 and 665 nm under inductive and noninductive conditions, respectively (*n* = 10; bars indicate standard errors of the means).

Once the identity of the compound produced by the *ebo* deletion mutants and mutant *ΔscyE* was confirmed, we sought to determine if, as can be predicted by its molecular structure, it would emit fluorescence upon excitation, allowing the investigation of its intracellular localization via fluorescence microscopy. Indeed, the scytonemin monomer exhibited a wide range of fluorescence emission in the blue spectrum with a maximum around 407 nm, in accordance with its absorbance spectrum (Fig. 4A inlay; excitation at 292 nm). We then tested if the levels of accumulation and the fluorescence yield would be sufficient for microscopy imaging. We used laser excitation at 405 ± 1 nm and collected emission at 410 ± 2 nm (rather than at the absolute maximum of 407 nm) to avoid excitation bleeding. Indeed, it was possible to visualize the accumulation of the scytonemin monomer in the mutants, as shown in an exemplary manner with confocal microscopy images for the wild-type, *Δebo*, and *ΔscyE* mutants, as well as in a quantitative manner for all mutants (Fig. 5). Wild-type N. punctiforme autofluorescence levels at 410 nm were low, both in induced and noninduced cells, but all mutants showed severalfold increases in fluorescence, as expected, upon induction and accumulation of the scytonemin monomer. In the absence of UVA induction, the fluorescence at 410 nm in all mutants was as basal as that of the wild type.

All microscopic images shown in Fig. 5 were obtained under identical microscope settings and, to avoid variability, stem from a single concurrent induction experiment. Inductions and microscopic imaging were replicated independently three times for each mutant. Autofluorescence of photosynthetic pigments at 665 nm is also shown for comparison. Some signs of chlorosis (content of photosynthetic pigments lower than that seen with the wild-type strain) were present in *ebo* and *ΔscyE* mutants under conditions of exposure to UVA radiation. In these experiments, periods of induction longer than 5 days resulted in obvious cellular damage (generalized loss of autofluorescence in both the blue and the red spectra), possibly due to photosensitization by the monomer under conditions of exposure to UVA radiation.

**FIG 6:**
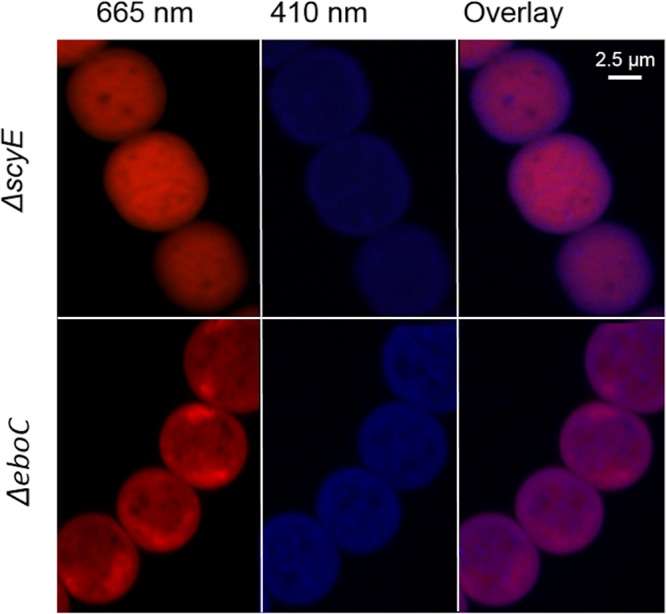
Intracellular localization of the scytonemin monomer in induced Δ*scyE* and Δ*eboC* cells. Overlay of the 665-nm images over the 410-nm images demonstrates the localization of the scytonemin monomer in the cytoplasm of Δ*eboC* cells (this was the case for each and all of the *ebo* mutants, as can be seen in Fig. S2). In Δ*scyE* cells; however, the scytonemin monomer accumulates in both the cytoplasm and the periplasm.

Because photosynthetic pigments are part of macromolecular complexes localized in the intracytoplasmatic thylakoid membranes (and sometimes also on the inner leaflet of the cytoplasmic membrane) (17), cell autofluorescence at 665 nm, which originates largely from chlorophyll *a* (Chl *a*) and phycobiliproteins, can be used to visualize the bounds of the cytoplasm. Having concurrent photopigment fluorescence data to define the bounds of the cytoplasm and bright-field images to establish the boundaries of the whole cell, we used comparative overlay images to determine the cellular localization of scytonemin monomer accumulation in the various mutants. The images clearly show the presence of the scytonemin monomer only in the cytoplasm in each of the *Δebo* cells but in both the cytoplasm and periplasm of the *ΔscyE* cells (Fig. 6; see also Fig. S3). Additionally, overlay of 410-nm emission and differential interference contrast images indicated that the scytonemin monomer was contained within the cell in *ΔscyE* cells (Fig. S4).

10.1128/mBio.02266-18.4 FIG S3 Localization of the scytonemin monomer in UVA-induced *ebo* mutants by overlay of 665-nm emission on the 410-nm emission images. Accumulation was cytoplasmatic in all cases. An ScyE deletion mutant is included for comparison. All scale bars are 2 μm. Download FIG S3, PDF file, 0.7 MB. Copyright © 2018 Klicki et al. 2018 Klicki et al. This content is distributed under the terms of the Creative Commons Attribution 4.0 International license.

10.1128/mBio.02266-18.5 FIG S4 Overlay images of 665-nm and 410-nm emission channels and differential interference contrast (DIC) images. For all mutants, fluorescence in the 410-nm channel was restricted to the cellular interior in all cases. Download FIG S4, PDF file, 0.2 MB. Copyright © 2018 Klicki et al. 2018 Klicki et al. This content is distributed under the terms of the Creative Commons Attribution 4.0 International license.

**FIG 7:**
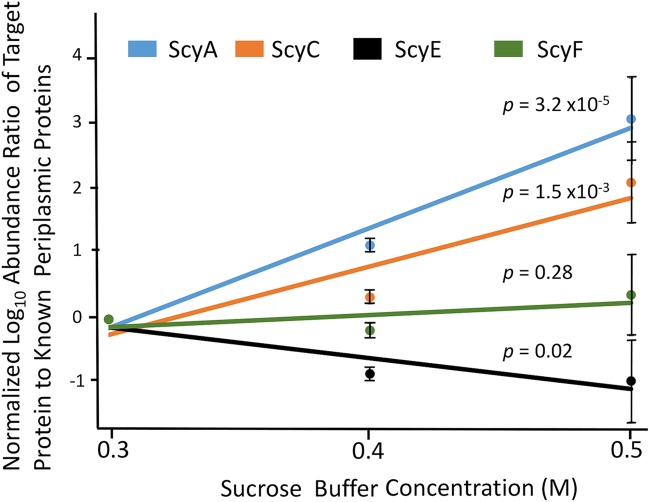
Partitioning of core biosynthetic proteins of the scytonemin operon between cytoplasm and periplasm by proteomic analyses of osmotic shock lysates. Target protein relative abundance ratios to three different known periplasm-targeted proteins are plotted against the strength of lysis buffer used. Proteins localized to the cytoplasm should show an increase in ratio with buffer strength, whereas ratios of proteins partitioning preferentially to the periplasm should remain invariant or decrease. All ratios were normalized to 1 at 0.3 M sucrose for ease of graphing, and *P* values are included for each target protein data set (α = 0.01).

In order to verify the periplasmic localization of ScyE suggested by its N-terminal Sec signal peptide, induced cultures were subjected to periplasmic fractionation. ScyE could be detected by proteomics in periplasmic lysate, as could other periplasmic proteins (S-layer domain protein, peptidase S8, and l-sorbosone dehydrogenase). The S-layer domain-containing proteins have been identified in the peptidoglycan layer of Gram-negative bacteria (18), necessitating their translocation to the periplasmic space after synthesis. l-Sorbosone dehydrogenase plays a role in l-sorbose assimilation, a periplasmic process (19). Ratios of abundances for cytoplasmatic proteins compared to those of the three standard periplasmic proteins should increase exponentially with increasing strength of osmotic shock due to dilution of the periplasmic contents by increased release of cytoplasm. This was demonstrably the case for cytoplasmatic ScyA and ScyC (Fig. 7). However, the abundance ratio of ScyE and ScyF to each of these periplasmic proteins either remained statistically invariant or decreased slightly with increasing strength of osmotic shock, indicating that they partitioned to the periplasm with consistency equal to or greater than that seen with the standard proteins (Fig. 7) and confirming the periplasmic localization of ScyE and ScyF.

In order to confirm the periplasmic localization of the scytonemin monomer with an approach other than microscopy, we assessed its differential release in periplasmic fractions of induced *ΔscyE* cells versus those in *Δebo* cells. We found that *ΔscyE* cells yielded on average 21% of the total cellular content of the scytonemin monomer to periplasmic preparations obtained with mild osmotic shock (0.2 to 0.3 M sucrose shock), whereas *Δebo* cells yielded only about 6%, which is consistent with a periplasmic localization of the scytonemin monomer in *ΔscyE* mutants and a cytoplasmic restriction in *Δebo* cells.

## DISCUSSION

The fact that every *ebo* deletion mutant resulted in a scytoneminless phenotype strongly suggested that the *ebo* genes are involved in the process of scytonemin production in N. punctiforme, despite their widespread occurrence in non-scytonemin-producing bacteria. This conclusion is consistent with their conserved placement within the scytonemin operon in most cyanobacteria (Fig. 3) and with their upregulation in N. punctiforme cells exposed to UVA radiation (9, 10). However, gene-specific phenol-indolic metabolites did not accumulate in *ebo* mutants, at least not to detectable levels, which does not fit the narrative suggesting that the *ebo* genes simply code for enzymes carrying out a sequential set of reactions involved in the late stages of scytonemin formation, as had been hypothesized previously (14). Rather, a single intermediary, the scytonemin monomer, accumulates in each of the *ebo* mutants under conditions of UVA induction (Fig. 1). This compound is identical to the combined products of ScyA-C when expressed heterologously in E. coli (15) and is just one oxidation step away from the product of ScyC *in vitro* (12), suggesting that the action of the Ebo proteins must be other than a fundamental biochemical transformation of the ScyC product. That all *ebo* knockout mutants resulted in the accumulation of the very same intermediary suggests that coordinated activity of all of the gene products is required. Thus, in entertaining the idea of a potential role for the *ebo* cluster that would satisfy the available biochemical evidence, one must envision an ancillary process, such as the synthesis of a cofactor necessary in the scytonemin synthesis pathway. Such a generic role is attractive in that the Ebo proteins could potentially serve similar roles in the various biochemical pathways of the bacterial and algal species in which they are found. But, as noted previously (16), the bioinformatics evaluation of the *ebo* gene products does not immediately suggest viable hypotheses as to what that role might be.

Because several of the scytonemin operon gene products had been predicted to be targeted to the periplasm (ScyDEF) since they possess canonical N-terminal signal peptides utilized by the Sec transport system (14), the late portion of the scytonemin biosynthesis was predicted to take place there, *en route* to eventual secretion (1, 10). Determining the cellular localization of the scytonemin monomer provided support for these predictions owing to the fact that its accumulation in *ebo* deletion mutants and mutant Δ*scyE* could be visualized by fluorescence microscopy. These investigations (Fig. 6) (see also Fig. S4 in the supplemental material) showed that in the absence of any one (or all) of the *ebo* genes studied, the scytonemin monomer remained restricted to the cytoplasm. By contrast, in mutant Δ*scyE*, where all of the *ebo* genes were intact, the monomer was also found in the periplasm, where it reached concentrations similar to those seen in the cytoplasm (Fig. S5). These results were confirmed by comparison of extraction yields of Δ*scyE* and Δ*ebo* periplasmic fractions. From this we deduce that the set of Ebo proteins plays a role in the translocation of the scytonemin monomer across the inner membrane, wherein the absence of any one of them prevents this translocation, leading to cytoplasmic accumulation. These findings also lead us to posit that ScyE is responsible for the oxidative dimerization of the scytonemin monomer to form the reduced form of scytonemin, with the process taking place in the periplasm, to which ScyE is demonstrably targeted. In mutant Δ*scyE*, the scytonemin monomer is excreted to the periplasm, only to find no enzyme target, and accumulates there, eventually causing feedback accumulation in the cytoplasm as well. In other mutants, such as those bearing deletions of ScyD or ScyF, representing proteins that are not central to biosynthesis, the scytonemin monomer is produced, exported, and further processed, thus leading to scytonemin-positive phenotypes. Taken together, our observations lead to a new model of scytonemin synthesis in which the *ebo* gene products act together for the translocation of the scytonemin monomer from the cytoplasm to the periplasm for final oxidative dimerization.

10.1128/mBio.02266-18.6 FIG S5 (A) Fluorescence intensity of the scytonemin monomer in cytoplasm and periplasm of *ΔscyE* cells. The periplasmic levels of accumulation of scytonemin monomer were comparable to those reached in the cytoplasm. (B) Fluorescence intensity of scytonemin monomer in cytoplasm and periplasm in *Δebo* cells. There was no significant accumulation of the scytonemin monomer in the periplasm. Fluorescence scattering or leakage may account for some low-level signal found outside the cytoplasm. Download FIG S5, PDF file, 0.2 MB. Copyright © 2018 Klicki et al. 2018 Klicki et al. This content is distributed under the terms of the Creative Commons Attribution 4.0 International license.

### A potential mechanism of action.

To entertain potential mechanisms for this translocation, we briefly review structural and phylogenetic traits of the proteins involved. *eboC* (Npun_F5234) presents strong homologies to the UbiA superfamily of prenyltransferases (16), transmembrane proteins that catalyze key prenylation steps in the production of various metabolites (20). EboC does indeed contain seven transmembrane domains (16) (see Fig. S6 in the supplemental material), being thus integral to the cytoplasmic membrane, and holds closest homology both to the digeranylgeranylglycerylphosphate (DGGGP) synthases involved in the synthesis of archaeal membrane lipids and to archaeal UbiA homologues involved in the synthesis of ubiquinone. Its predicted active site (Fig. S6) contains both the cluster of 4 asparagine residues that interact with the prenyl-group donor and the residues (Arg142 and Asp145) responsible for prenyl acceptance (21). We did not analyze *eboD* because it is not present in the N. punctiforme *ebo* cluster proper, but a homologue is found nearby in the opposite reading direction, and it is generally integrated within the *ebo* operon in other bacteria. EboD presents clear homology with sugar phosphate cyclases and has been posited to act on sedoheptulose-7-P (16). EboA has no close sequence homologs, but a structural prediction reveals several repeating alpha helices (Fig. S6), consistent with the tetratrico repeat (TPR) domain responsible for protein-protein complex stabilization (22, 23). Among the *ebo* gene products, only EboC and EboB contain parallel alpha-helices reminiscent of a TPR domain; thus, EboA may facilitate formation of an EboCAB complex anchored to the cell membrane, where the *eboA* knockout mutant fails to organize the correct assembly. Assigning a potential role to the other *ebo* genes becomes much more speculative. *eboB* is annotated as encoding a putative hydrolase, representing an enzyme class with diversity sufficient to preclude further predictions. *eboE* (Npun_F5235) codes for a triosephosphate isomerase (TIM) barrel-containing enzyme annotated as a putative xylose isomerase, and *eboF* (Npun_F5236) shares homology with pyrophosphatases. These considerations suggest that the enzymatic reactions carried out by the *ebo* cluster genes include the modification of sugars, likely including cyclitol formation from heptose precursors, and the prenylation of an undetermined substrate. Prenylated molecules, so-called lipid carriers, are indeed known as integral components of two other periplasmic metabolite translocation processes in bacteria: the synthesis of cell wall peptidoglycan (24) and the excretion of capsular polysaccharides (CPS)/lipopolysaccharides (LPS) (25). In the latter case, the carrier is a glycolipid that is translocated along with the polysaccharide chain through a transmembrane ATP-binding-cassette (ABC) transporter protein complex (25). The carrier is composed of a lyso-phosphatidylglycerol moiety and the linker sugar 3-deoxy-d-o-oct-2-ulosonic acid (or keto-deoxyoctulosonate [KDO]) (25). Thus, one can see some parallels between a potential *ebo* cluster product composed of a sugar cyclitol and a lipid carrier similar to that described for CPS systems, as this would enable the translocation of the scytonemin monomer using the existing CPS ABC permease. Indeed, scytonemin synthesis and CPS excretion are related processes in *Nostoc*; UVA elicits the production of both (26), and *Nostoc* mutants deficient in scytonemin production display enhanced capsular polysaccharide production under conditions of UVA induction relative to the wild type (7), as if the two processes were competing in the wild type. In fact, the *Nostoc* gene with the closest homology to a well-described CPS ABC permease (Npun_R5235) is among those upregulated by UVA exposure in global transcriptomic studies (27). Considering that no lipid-conjugated scytonemin monomer intermediate was found in the HPLC-mass spectrometry (HPLC-MS) analyses performed in this study, it is not likely that covalent bonding is responsible for association of the putative *ebo* lipid carrier and the scytonemin monomer. Perhaps noncovalent interactions such as polar-π bonding (28) between hydroxyls of the sugar head group of the carrier and the aromatic rings of the monomer facilitate this attachment. In any event, investigation of the accuracy of this proposed mechanistic model will constitute a long-term task that may benefit from a molecular characterization of the *ebo* proteins and their products as they pertain to glycolipid biosynthesis rather than indole-phenol intermediaries, as investigated here. We find this model of *ebo* function an attractive hypothesis for future testing, given its integration with a well-defined and ubiquitous membrane translocation system as well as its consistency with CPS formation.

10.1128/mBio.02266-18.7 FIG S6 I-TASSER (37) predicted structures of EboA and EboC. (A) Cartoon representation of EboA showing a putative repeating alpha-helix motif consistent with the structure of the solved TPR domain-containing peptides. (B) Surface rendering of EboA. (C) Cartoon representation of EboC showing putative transmembrane domains formed by six alpha helices in a cylindrical organization. (D) Surface rendering of EboC showing the substrate-accepting pocket containing basic residues, highlighted with the yellow circle. (E) Predicted structure of EboC shown in cartoon format angled towards the prenyl donor substrate-accepting pocket. (F) Surface rendering of EboC predicted structure, with the prenyl acceptor pocket highlighted with a yellow oval. Download FIG S6, PDF file, 0.1 MB. Copyright © 2018 Klicki et al. 2018 Klicki et al. This content is distributed under the terms of the Creative Commons Attribution 4.0 International license.

10.1128/mBio.02266-18.8 FIG S7 Gel electrophoresis of reverse transcriptase PCR assays in induced *ebo* mutants. (Top) Primer set (see Table S2) used to amplify *ebo* transcript cDNA. (Middle) *ebo* knockout mutant tested shown in gel lane below. (Bottom) Corresponding stained-gel images. Below the gel images is a genomic map of primer binding sites in the context of the *ebo* gene cluster and the corresponding PCR product size. Download FIG S7, PDF file, 0.1 MB. Copyright © 2018 Klicki et al. 2018 Klicki et al. This content is distributed under the terms of the Creative Commons Attribution 4.0 International license.

### Roles of *ebo* genes beyond cyanobacteria.

As a recognizable genomic element in the genomes of over 150 microbes that occupy diverse ecological niches, the impact of the *ebo* cluster clearly exceeds the biology of scytonemin and cyanobacteria. The present findings represent the first description of *ebo* gene function in any organism, potentially shedding light on the reason for their high evolutionary conservation across disparate phyla of bacteria and algae. In the literature to date, there has been only one tangential study of the *ebo* gene cluster function. Burlinson et al. (29) produced transposon insertion mutants in a cluster of Pseudomonas fluorescens NZ17 that rendered the bacterium vulnerable to predation by the nematode Caenorhabditis elegans; they hence named this genetic region the EDB (for “edible”) cluster and conjectured that it codes for the synthesis of a nematode repellant compound. The EBD cluster in fact contains a full *ebo* cluster, in which transposon-interrupted *eboA*, *eboD*, or *eboE* homologues result in the edible phenotype. Furthermore, the repellant compound was not found in NZ17 supernatant, suggesting capsular localization similar to that seen with scytonemin. Interestingly, some of the other non-*ebo* EBD open reading frames contain signal peptides for excretion to the periplasm, and genes homologous to the CPS ABC permease systems are found just upstream of the EDB cluster. With these coincidences and the hindsight of our study, it is fair to postulate that the *ebo* homologues within the EDB cluster may very well facilitate the excretion of such a hypothetical nematode repellant in *Pseudomonas*. A generic role in metabolite excretion for the *ebo* genes may also offer explanations for their relative incidences in symbiotic interactions between bacteria and microalgae (16, 30).

## MATERIALS AND METHODS

### Cultures and culture conditions.

All experiments were conducted with a wild-type Nostoc punctiforme strain ATCC 29133 (PCC 73102) derivate, UCD 153, that displays dispersed growth and a higher frequency of gene replacement by conjugal transfer (31) and with mutants derived from it (see Table S1 in the supplemental material). All strains of N. punctiforme were grown as previously described (14) in liquid Allen and Arnon medium (32), diluted 4-fold (AA/4), and on solidified AA medium plates. When necessary, the medium was supplemented with 2.5 mM NH_4_Cl buffered with 5 mM MOPS (morpholinepropanesulfonic acid) (pH brought to 7.8 by dropwise addition of NaOH). Neomycin was used at 25 µg ml^−1^ for the selection and maintenance of transformed single recombinants. Escherichia coli strains and derivatives were grown in liquid or solid lysogeny broth (LB) (33) supplemented with kanamycin at 25 µg ml^−1^ and, when required, with chloramphenicol at 30 µg ml^−1^.

### Construction of mutants.

All chromosomal mutations in this study were in-frame deletions of individual genes, with the exception of Δ*ebo*, where the entire five-gene cluster was deleted. The deletions were generated by PCR using N. punctiforme genomic DNA and primers designed to amplify DNA upstream and downstream of the deletion (2.0 kb to 3.0 kb on each side to allow for homologous recombination), with the primers adjacent to the deletion containing overlapping sequences (see Text S1 and Table S1 in the supplemental material) (38, 39).

10.1128/mBio.02266-18.1 TEXT S1 Additional materials and methods. Download Text S1, PDF file, 0.2 MB. Copyright © 2018 Klicki et al. 2018 Klicki et al. This content is distributed under the terms of the Creative Commons Attribution 4.0 International license.

### Screening for polar transcriptional effects.

In order to verify that the *ebo* gene in-frame deletion mutations did not deleteriously affect transcription of downstream *ebo* genes, RT-PCR was used. For each mutant, we targeted the transcript of the next gene downstream under inductive conditions, except for the *ΔeboF* mutant, where downstream effects would be irrelevant. For this, total RNA was extracted using a Mobio Powersoil total RNA extraction kit, from which cDNA was synthesized using Superscript III reverse transcriptase (Thermo Fisher Scientific). Primers specific to *eboB*, *eboC*, *eboE*, and *eboF* (Table S2) were used to amplify cDNA fragments corresponding to their parent mRNA. The resulting PCR products were analyzed by gel electrophoresis against reactions run with wild-type genomic DNA as a synthesis template.

10.1128/mBio.02266-18.10 TABLE S2 Primers used in this work for RT-PCR assessment of polar transcriptional effects. Download Table S2, PDF file, 0.2 MB. Copyright © 2018 Klicki et al. 2018 Klicki et al. This content is distributed under the terms of the Creative Commons Attribution 4.0 International license.

### Biochemical characterization of mutant strains.

Cells from N. punctiforme wild-type and derived deletion mutants were tested for their ability to produce scytonemin upon induction by UVA radiation as previously described (7). Following UVA exposure, the cells were harvested and the lipid-soluble pigments were extracted in equal volumes of 100% acetone. Extracts were initially analyzed spectrophotometrically between 330 nm and 730 nm, with a strong absorption peak at 384 nm indicating that scytonemin had accumulated in the cells (3). Following UVA exposure, water-soluble compounds were also extracted from whole cells in equal volumes of 25% aqueous methanol. A 50-µl volume of concentrated acetone or methanol extracts from cells exposed to UVA radiation was also analyzed by HPLC (see Text S1) (40). Unknown peaks of interest were sourced from five independent biological replicates and collected with a Gilson FC 205 fraction collector. Exact masses of collected compounds were analyzed by electrospray ionization mass spectrometry (MS) using a Bruker Daltonics micrOTOF-Q instrument in positive- and negative-ion modes. Fractions collected from the wild-type strain at the same retention time and solvents utilized to run the HPLC experiments were used as negative controls for MS. Authentic standards for the scytonemin monomer (Fig. 2) were obtained by heterologous expression of *scy* genes in E. coli following the method described by Malla and Sommer (15) (see Text S1). The scytonemin monomer (Fig. 1) was purified and characterized with respect to its absorbance spectrum, retention time, and mass to serve as an authentic standard. Additionally, steady-state fluorescence spectra of the scytonemin monomer were obtained using a LS-55 fluorescence spectrometer (PerkinElmer Inc., Waltham, MA) equipped with a red-sensitive R928 photomultiplier tube (PMT) detector (Hamamatsu Corporation, Bridgewater, NJ). The collected compound was diluted in HPLC-grade acetonitrile in a 1-cm-path-length quartz cuvette to an optical density at 292 nm of 0.2. Emission spectra were determined at room temperature and were analyzed at between 300 and 555 nm in 0.5-nm increments with 292-nm excitation. One hundred spectra were collected and averaged to reduce noise, and the spectrum of acetonitrile alone was collected to confirm that solvent did not contribute to the sample spectrum. Finally, the spectrum was corrected for the sensitivity profile of the detector using a manufacturer-supplied correction file.

### Cellular characterization of mutant strains.

To determine the intracellular localization of the accumulated scytonemin monomer, we used fluorescence confocal microscopy. UVA-induced wild-type and Δ*scyE* strains and all *ebo* gene deletion strains were cultured and treated as described previously (7). After 5 days of exposure to UVA, cells were collected and wet mounts were prepared and imaged on a Leica TCS SP5 AOBS spectral confocal system, using both bright-field and fluorescence microscopy. Laser excitation was at 405 nm. Emission at 665 nm was used to visualize photosynthetic pigment fluorescence (chlorophyll *a* and phycobilin emission), and emission at 410 nm was used to visualize scytonemin monomer fluorescence. All images were taken at ×400 magnification. Fluorescence quantification and image analyses were performed using ImageJ (34). Additional imaging was carried out using a Zeiss LSM800 laser scanning confocal microscope equipped with a Plan-Apochromat 63 by 1.40 numerical aperture (NA) oil immersion objective with fluorescence excitation wavelengths as described above. Further contrast adjustment for presentation purposes was done using Zen 2.3 (Carl Zeiss Microscopy GmbH, 2011) image analysis software.

### Cellular localization of ScyE.

Bioinformatic analysis of ScyE revealed that it contains an N-terminal Sec pathway signal peptide, suggesting that it is periplasmically localized. To confirm this prediction, UVA-induced and uninduced wild-type N. punctiforme cultures (*n* = 6) were harvested and subjected to lysis by osmotic shock as described previously by Ross et al. (35) with the following revisions: 300 mM sucrose lysis buffer was used to preferentially lyse the outer membrane, while increasingly efficient whole-cell lysates were obtained using 400 and 500 mM lysis buffers. Proteomic analyses of lysate preparations were then conducted following Mitchell et al. (36). In order to ascertain cellular localization of gene products of interest, we compared the ratios of the abundances (normalized spectral abundance factor [NSAF]) of all of the relevant proteins (ScyA, ScyC, ScyE, and ScyF) to the corresponding abundances (NSAF) of three known periplasm targeted peptides (S-layer domain protein, peptidase S8, and l-sorbosone dehydrogenase; Uniprot accession numbers B2J6K1, B2JAL9, and B2IVQ5, respectively). The ratios were plotted against the molar concentration of lysis buffer, and the exponential regressions were assessed by analysis of variance (ANOVA).

### Presence of the scytonemin monomer in periplasmic extracts.

To confirm the differences in cellular periplasmic localization of the scytonemin monomer in the *ΔscyE* versus *Δebo* mutants, we assessed the susceptibility of its extraction from cell lysates induced by mild osmotic shock. Induced *ΔscyE* and *Δebo* cells were shocked as described above (35) and the lysates (3 ml) extracted by mixing in equal volumes of ethyl acetate, followed by phase separation. The lipid-soluble extracts were analyzed by HPLC as described above, and the scytonemin monomer was quantified fluorometrically, with excitation at 293 nm and emission detected at 407 nm. Percentages of yields of lysate fractions were compared to those determined for the total extracts from cell pellets in the corresponding preparations.
