# Supplementary material for: The Widely Conserved ebo Cluster Is Involved in Precursor Transport to the Periplasm during Scytonemin Synthesis in Nostoc punctiforme
Source: mBio. 2018 Nov 27;9(6):e02266-18. doi: 10.1128/mBio.02266-18 (PMC6282210; doi:10.1128/mBio.02266-18)
Supplement: TABLE S1 [file mbo006184193st1.pdf]

Supplementary Table S1

Table S1 A. *N. punctiforme* strains and oligonucleotides used in this work.

N. punctiforme strains	Relevant characteristics and details of mutants construction	Oligonucleotides used to confirm homozygosity of mutants
WT	UCD 153: ATCC 29133 wild-type derivate, scytonemin producer (Campbell *et al.*, 2007)	
Δ*scyE*	scytoneminless mutant (Ferreira and Garcia-Pichel, 2016)	
Δebo	Npun_F5232 to Npun_F5236 knockout. Primers used to create a 5364 bp deletion:<br>sat-XhoI: ATCATCCTCGAGCGACAAATCCACCTGGTGGACTTC<br>satI1: ACGCGGTACAGCACTAAATGCC<br>satI2: GGCATTTAGTGCTGTACCGCGTGGCTCTCACGGTAATATCACCAC<br>satR: CCCGACTTCTTCAAGAAATCGGG	satF: AAGTTGGGCAACTTCTCCTG<br>satR2: GCTGGATAAGGGTTACGTAG<br>satF2: GGGTAAACAAGCCTACCATC<br>F5234R<br>satF3: GCCAAACTGGGCAGATGAAAGC<br>F5232R
Δ*eboA*	Npun_F5232 knockout. Primers used to create a 606 bp deletion:<br>sat-XhoI<br>satI1<br>F5232I2: GGCATTTAGTGCTGTACCGCGTTGTGCCGAGTCTCCTTTACC<br>F5232R: GCCGATGGAATCCACCATAAGG	satF<br>F5233I1<br>satR2<br>F5232F: CTGTAGGGCGGTTTGCGGATAG
Δ*eboB*	Npun_F5233 knockout. Primers used to create a 720 bp deletion:<br>sat-XhoI<br>F5233I1: CGTAAGTAGTACGGGAACAC<br>F5233I2: GTGTTCCCGTACTACTTACGGCCAAACTGGGCAGATGAAAGC<br>F5233R: ACGCGCAAACACAGATTGTGG	F5232F<br>F5234I1<br>F5233R2: AAGCGTTCCCAGCCTACAAGAC<br>F5235L-XhoI
Δ*eboC*	Npun_F5234 knockout. Primers used to create a 657 bp deletion:<br>F5234L-XhoI: ATCGCGCTCGAGAAGTTGGGCAACTTCTCCTG<br>F5234I1: AGCCGGAAGCAGCGAAACCTAC<br>F5234I2: GTAGGTTTCGCTGCTTCCGGCTTAGCCGGAAATATCCGAAATGCG<br>F5234R: CAGCCTCTATACTGCCTTTC	satF3<br>F5232R<br>F5234R2: CCGCCGCCGTATAAACCACTTG<br>F5234F: CGATCGCAGCACTACCATTCG
Δ*eboE*	Npun_F5235 knockout. Primers used to create a 768 bp deletion:<br>F5235L-XhoI: ATCGCGCTCGAGGCTAGTCAGTTTGGCATCCAACAC<br>F5232R<br>F5235I2: CCTTATGGTGGATTCCATCGGCCTACCCAAGATGACATCGCTACTG<br>F5235R: CTGAATACACATCCGCCACTTAGG	F5234F<br>F5236I1<br>F5233R<br>F5235F: ACGTCGCACTGATGGTACACTG
Δ*eboF*	Npun_F5236 knockout. Primers used to create a 1131 bp deletion:<br>F5236L-XhoI: ATCGCGCTCGAGGGGCGGATATTCTTGTAGGTTTCG<br>F5236I1: ATTGACGCTACCTGTCCTTTCG<br>F5236I2: CGAAAGGACAGGTAGCGTCAATTGGCTCTCACGGTAATATCAC<br>satR	F5235F<br>F5234R<br>F5236R2: GCGACAATCCCATGTTCATC<br>satF2

Underlined letters indicate introduced XhoI restriction endonuclease cleavage site.

Table S1 B. *E. coli* strains and oligonucleotides used in this work

E. coli strains	Relevant characteristics and details of mutant construction	Oligonucleotides used to confirm mutants
E. coli harboring pRSFDuet:*scyABC*	Forward primer containing NcoI restriction site:<br>5'-GCCGCCCATATGACTATACTGGTTCC-3'<br>Reverse primer containing SacI restriction site:<br>5'-TTAGTTGGGAACTAGGGATTCTTG-3'	5'-GCCGCCCATATGACTATACTGGTTCC-3'
E. coli harboring pRSFDuet:*scyABC-scyEF*	Forward primer containing NdeI restriction site<br>5'-TGGGATTCGTTGTCTTTAAACCC-3'<br>Reverse primer containing FseI restriction site<br>5'-TCAGCATTGTTGCTTTTGCAGTTCTTTC-3'	5'- TGGGATTCGTTGTCTTTAAACCC-3'
