# Supplementary figures and images for: The Widely Conserved ebo Cluster Is Involved in Precursor Transport to the Periplasm during Scytonemin Synthesis in Nostoc punctiforme
Source: mBio. 2018 Nov 27;9(6):e02266-18. doi: 10.1128/mBio.02266-18 (PMC6282210; doi:10.1128/mBio.02266-18)

**A**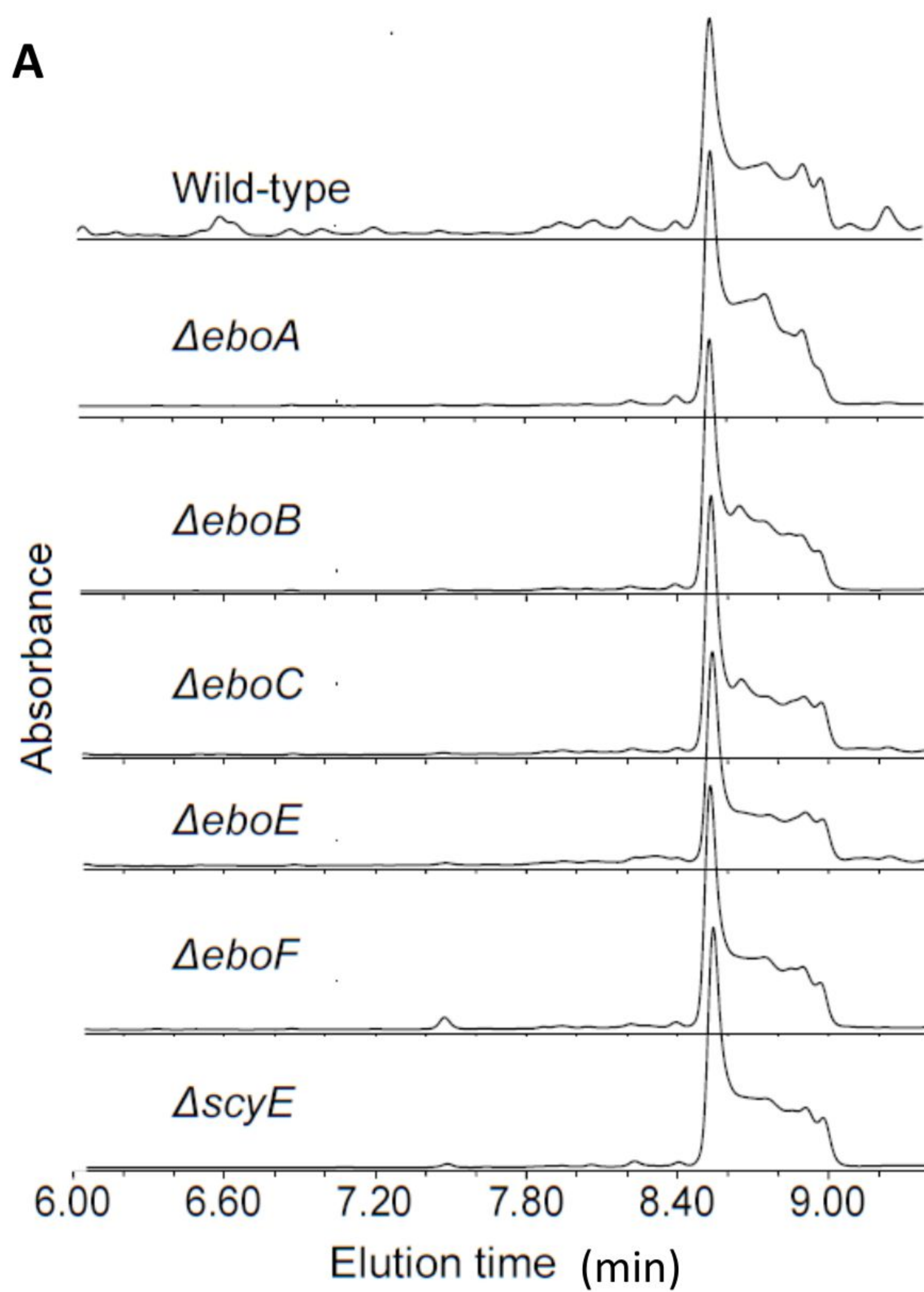**B**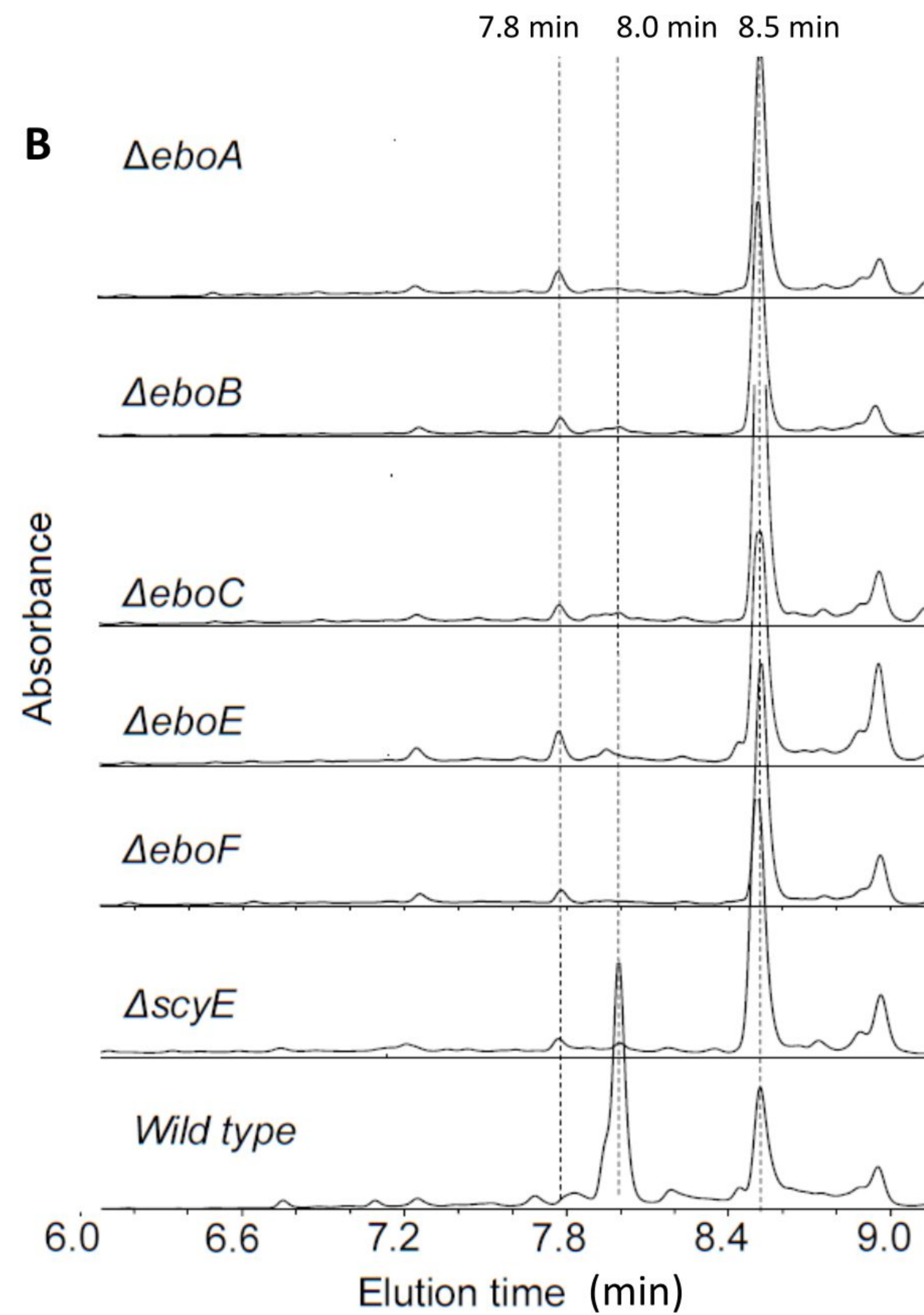

Supplement: FIG S1 [file mbo006184193sf1.pdf]

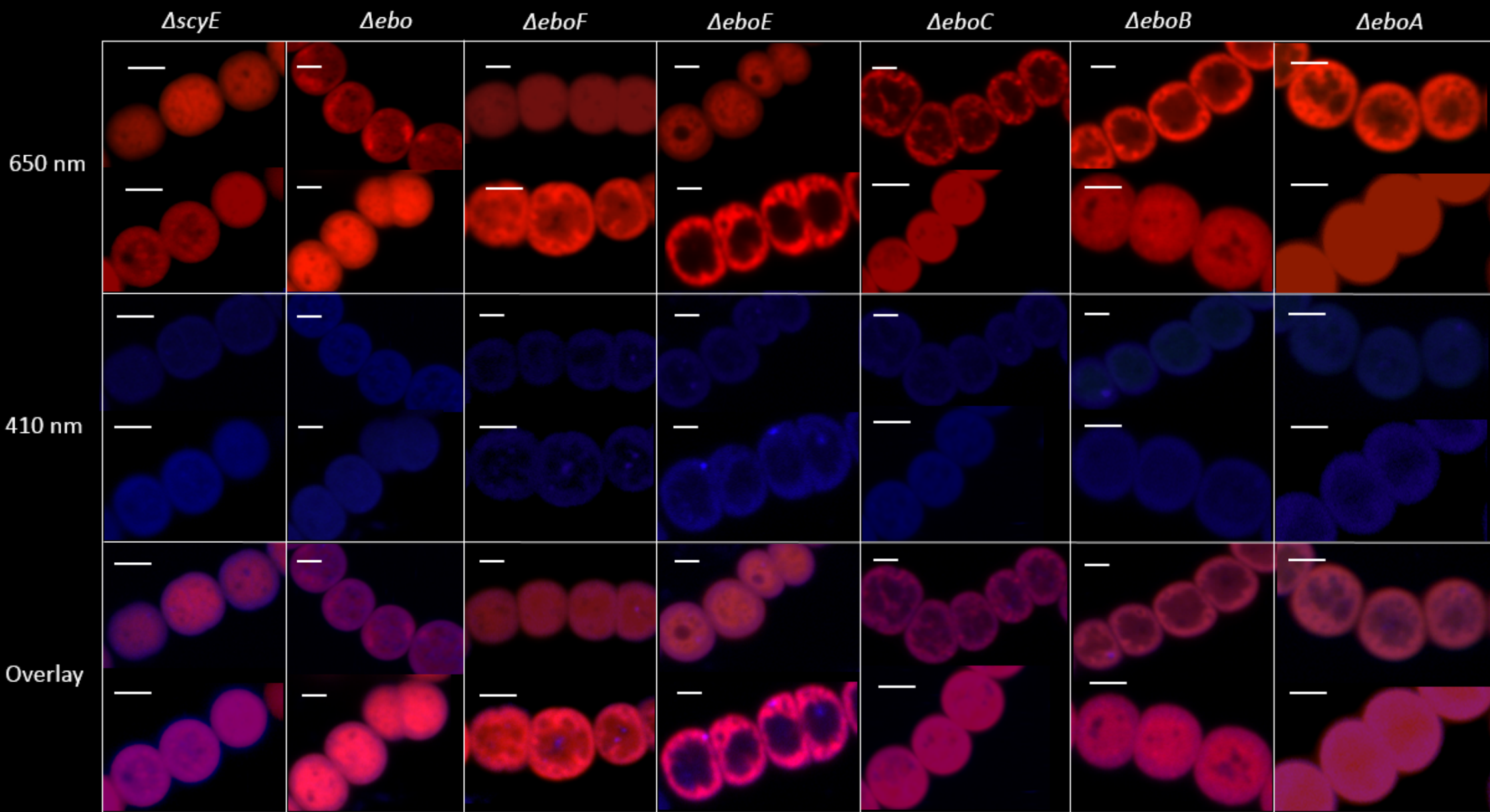

Supplement: FIG S3 [file mbo006184193sf3.pdf]

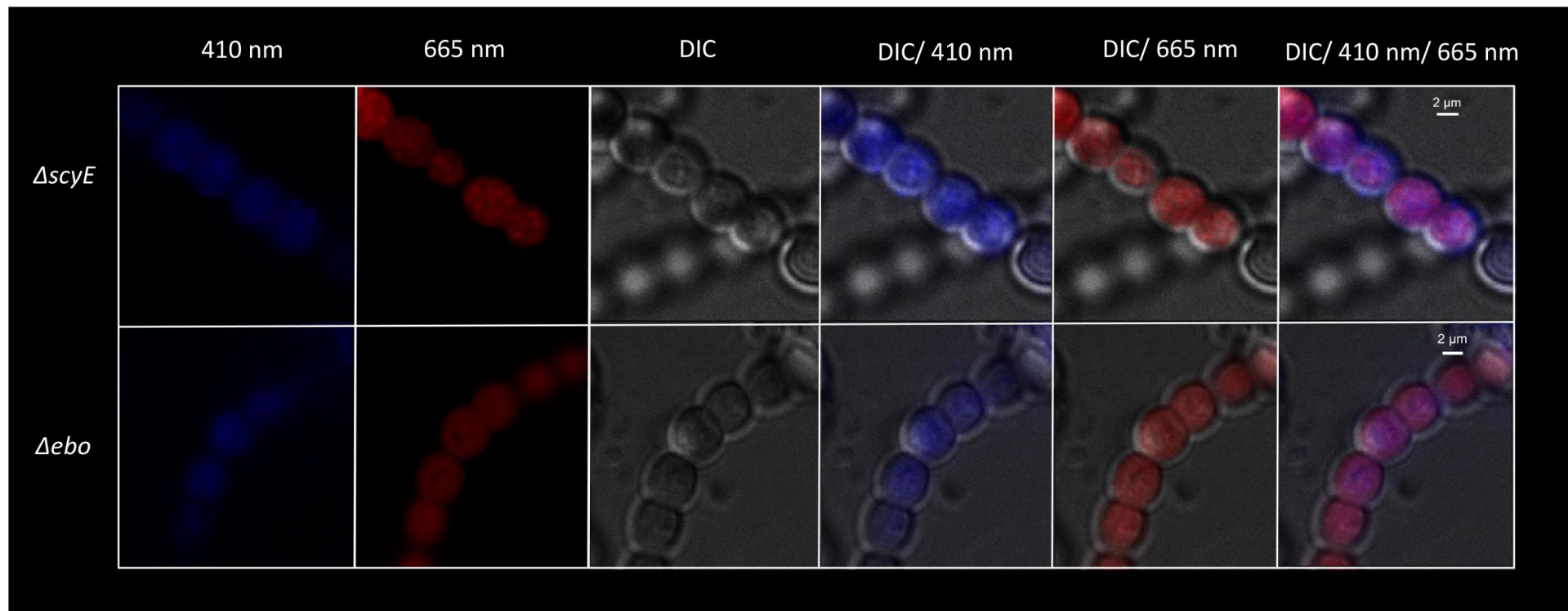

Supplement: FIG S4 [file mbo006184193sf4.pdf]

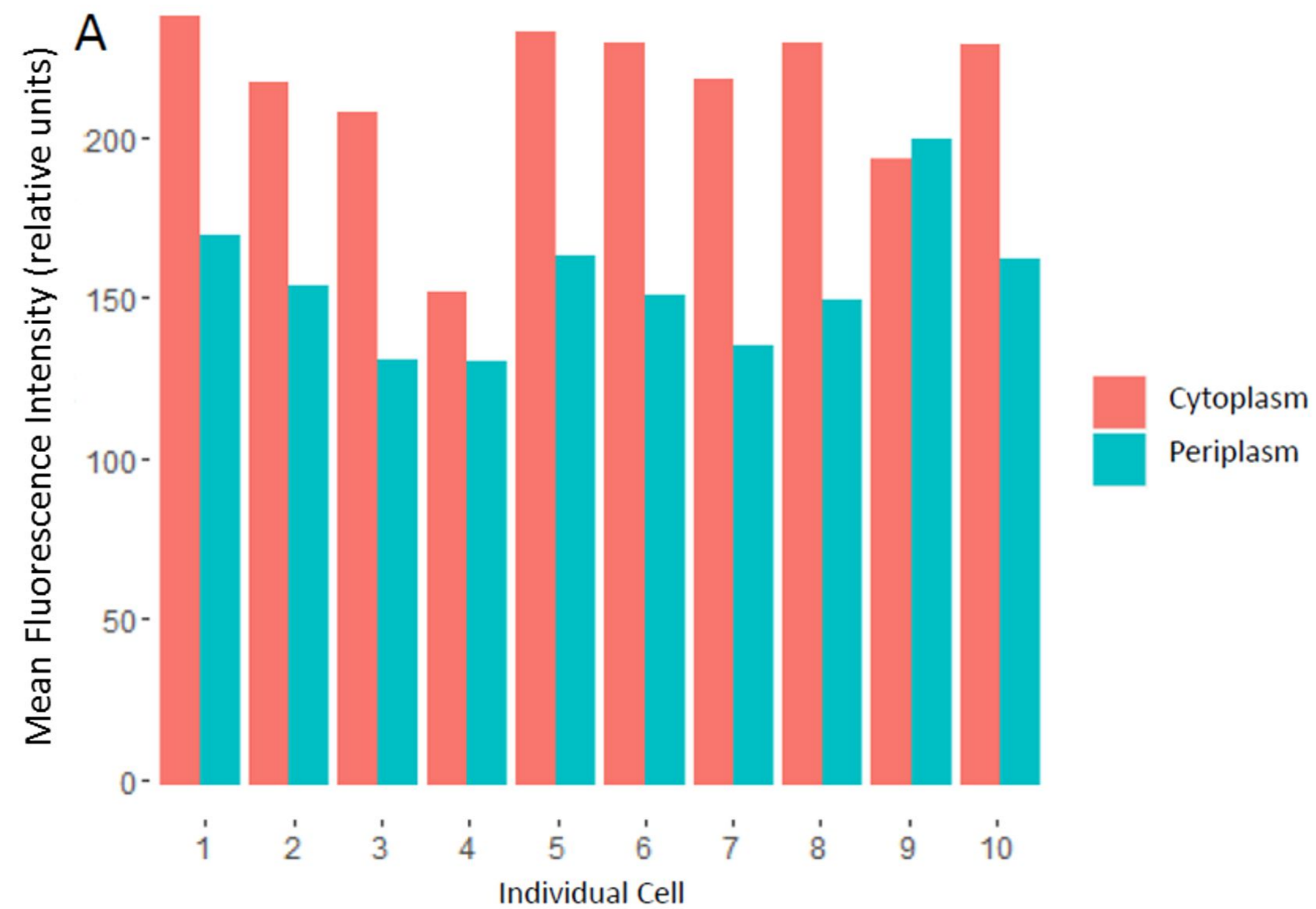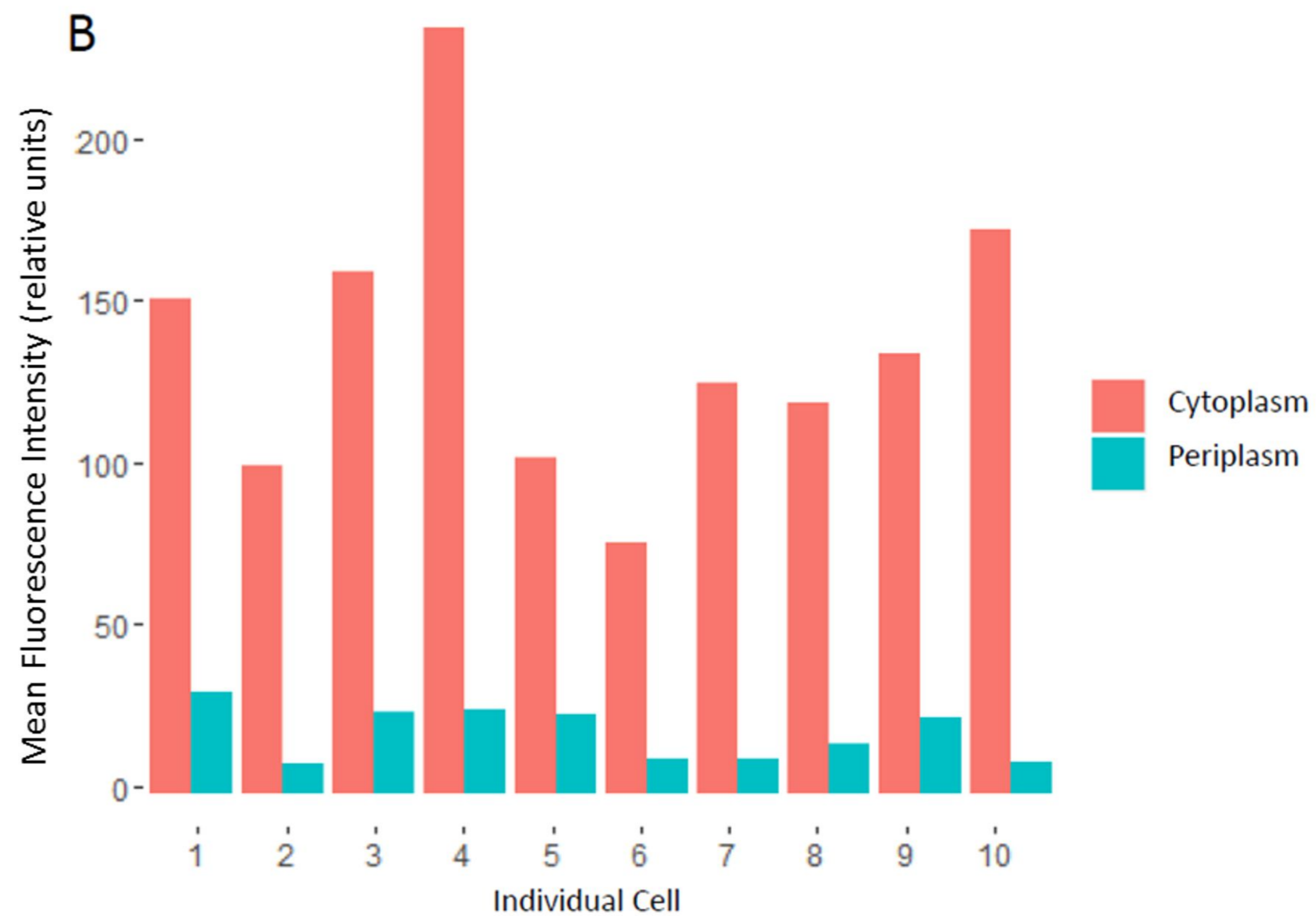

Supplement: FIG S5 [file mbo006184193sf5.pdf]

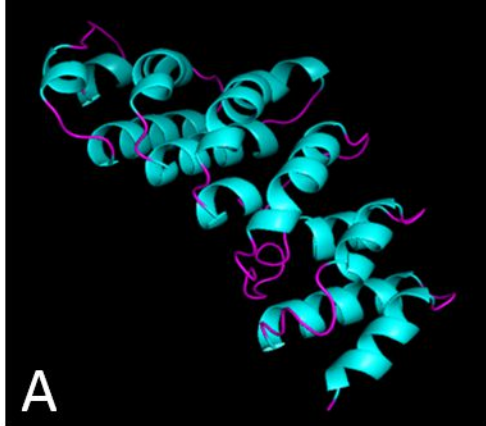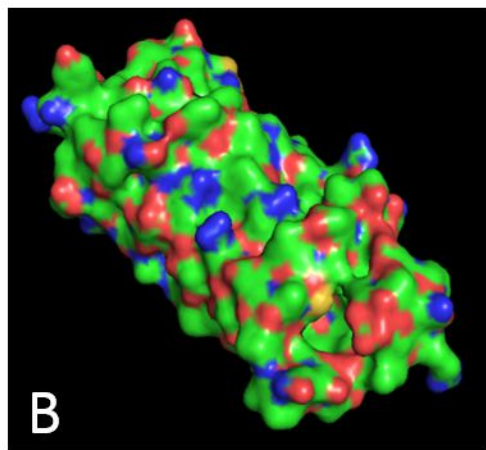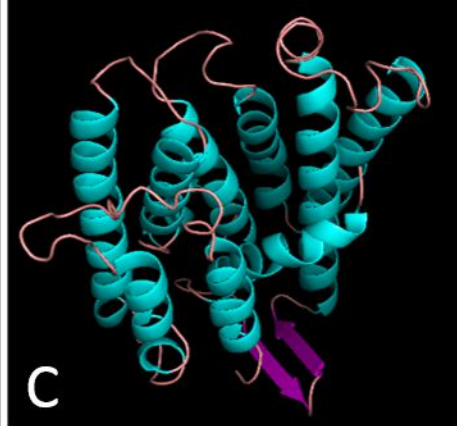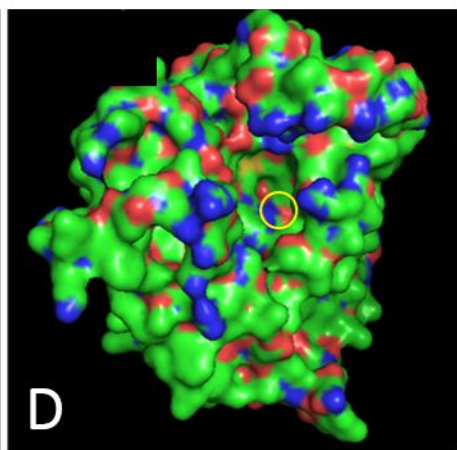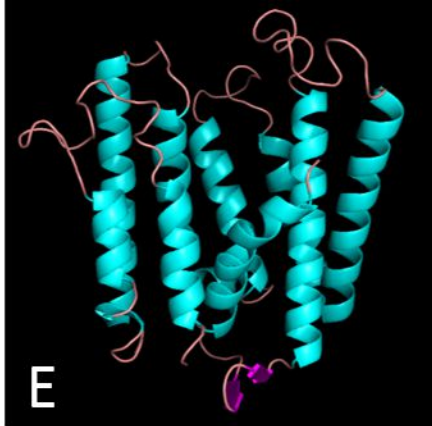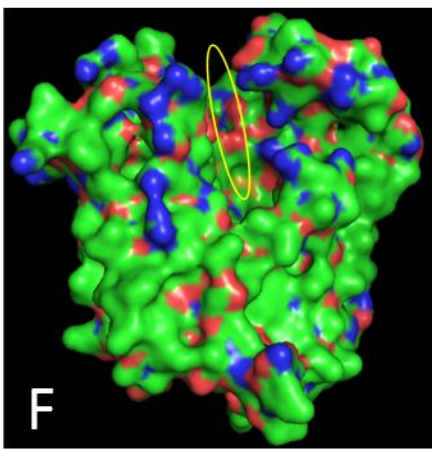

Supplement: FIG S6 [file mbo006184193sf6.pdf]
