# Supplementary material for: The Widely Conserved ebo Cluster Is Involved in Precursor Transport to the Periplasm during Scytonemin Synthesis in Nostoc punctiforme
Source: mBio. 2018 Nov 27;9(6):e02266-18. doi: 10.1128/mBio.02266-18 (PMC6282210; doi:10.1128/mBio.02266-18)
Supplement: FIG S2 [file mbo006184193sf2.pdf]

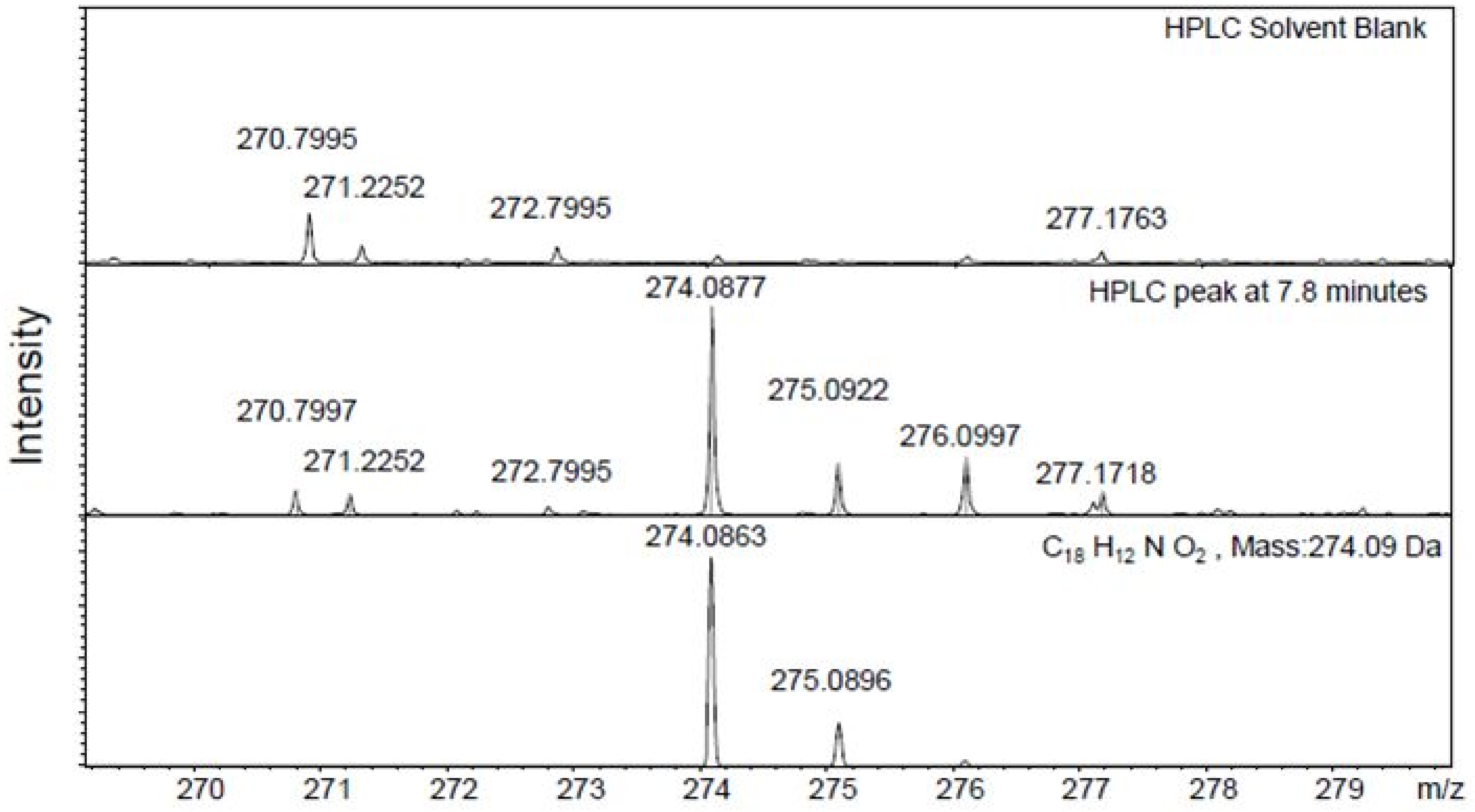
HPLC Solvent Blank
270.7995
271.2252
272.7995
277.1763
HPLC peak at 7.8 minutes
274.0877
275.0922
270.7997
276.0997
271.2252
272.7995
277.1718
274.0863
$C_{18}H_{12}NO_2$, Mass:274.09 Da
275.0896
Intensity
270
271
272
273
274
275
276
277
278
279
m/z
