# Supplementary material for: The Widely Conserved ebo Cluster Is Involved in Precursor Transport to the Periplasm during Scytonemin Synthesis in Nostoc punctiforme
Source: mBio. 2018 Nov 27;9(6):e02266-18. doi: 10.1128/mBio.02266-18 (PMC6282210; doi:10.1128/mBio.02266-18)
Supplement: FIG S7 [file mbo006184193sf7.pdf]

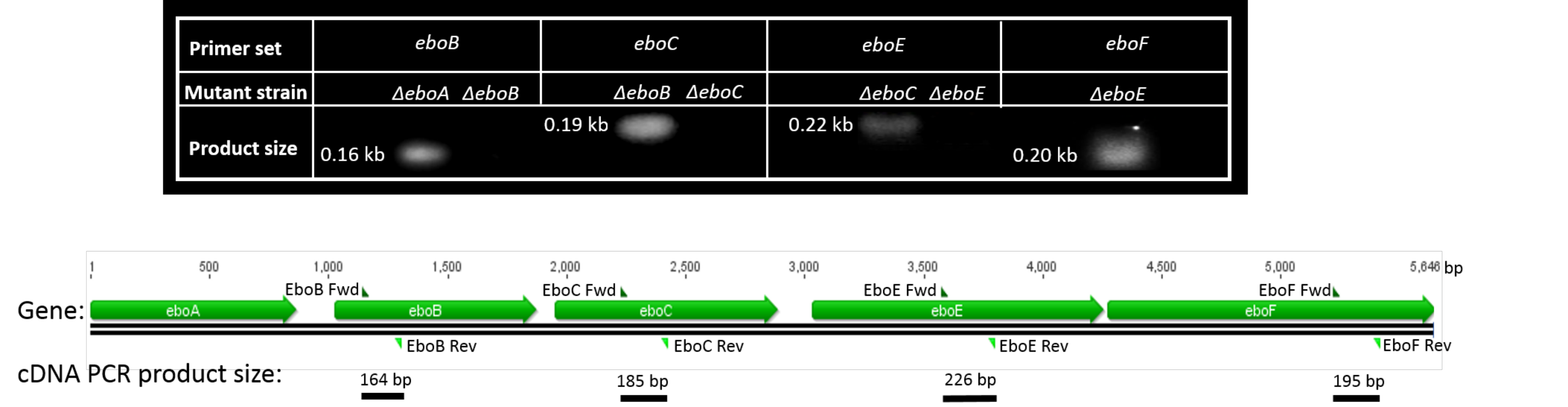

Primer set
eboB
eboC
eboE
eboF
Mutant strain
ΔeboA ΔeboB
ΔeboB ΔeboC
ΔeboC ΔeboE
ΔeboE
Product size
0.16 kb
0.19 kb
0.22 kb
0.20 kb
1
500
1,000
1,500
2,000
2,500
3,000
3,500
4,000
4,500
5,000
5,646 bp
Gene:
EboB Fwd
EboC Fwd
EboE Fwd
EboF Fwd
eboA
eboB
eboC
eboE
eboF
EboB Rev
EboC Rev
EboE Rev
EboF Rev
cDNA PCR product size:
164 bp
185 bp
226 bp
195 bp
