# Supplementary material for: The Widely Conserved ebo Cluster Is Involved in Precursor Transport to the Periplasm during Scytonemin Synthesis in Nostoc punctiforme
Source: mBio. 2018 Nov 27;9(6):e02266-18. doi: 10.1128/mBio.02266-18 (PMC6282210; doi:10.1128/mBio.02266-18)
Supplement: TEXT S1 [file mbo006184193s1.pdf]

Supplementary Materials and Methods

Construction of mutants. All chromosomal mutations were in-frame deletions generated by PCR using *N. punctiforme* genomic DNA. Primers were designed to amplify DNA to the right and left of the deletion (≥2.0 kb on each side to allow for homologous recombination) with the primers adjacent to the deletion containing overlapping sequences (see Supplementary Table S1 for oligonucleotide details and gene deletions). PCR products from these reactions were mixed and allowed to anneal via overlapping sequences. Subsequent PCR yielded a product containing the deletion, which was then cloned into the *sacB*-containing plasmid pRL278 [(18)] as a XhoI fragment using restriction sites contained within the primers. The resulting recombinant plasmids were sequenced to ensure fidelity. Gene deletion suicide-plasmids were introduced into wild-type *N. punctiforme* via triparental conjugation using *E. coli* strains as carriers of recombinant plasmids according to [(19)], with two variations from the original protocol: no sonication of *N. punctiforme* prior to conjugation, and no supplementation of conjugative plates with CO_2-enriched air. Single recombinant strains, in which the suicide-plasmid was integrated into the genome, were selected and maintained in medium containing neomycin. Strains in which the suicide-plasmid and wild-type gene were eliminated via a second recombination event, were selected by the presence of 5% (w/v) sucrose, wherein presence of *sacB* lead to the production of toxic levan compounds from the sucrose, forcing counterselection against the *sacB* containing suicide plasmid. PCRs performed as previously described [(12)] confirmed elimination of the wild-type gene and in-frame replacement with the respective 3’ and 5’ ends of the inactivated gene (see supplementary Table S1 for oligonucleotide details). In addition to new mutants obtained in this work, we also analyzed deletion mutants Δ*scyD,* Δ*scyE*, and Δ*scyF*, described previously [(12)].

Biochemical characterization of mutant strains. Cells from *N. punctiforme* wild-type and derived deletion mutants were tested for their ability to produce scytonemin upon induction by UVA radiation. Cultures grown for five days, with a chlorophyll *a* content of approximately 1–2 μg ml^{-1}, were concentrated to 50 μg of chlorophyll *a* ml^{-1} and spread over polycarbonate membrane filters (0.4 μm pore size), which were placed floating on liquid Allen and Arnon medium filled glass petri dishes, as previously described [(2)]. The cells were exposed to white fluorescent light (7 Wm^{-2}) supplemented with UVA radiation (10 Wm^{-2}) as described previously [(12)], continuously for five days. Following UVA exposure, the cells were harvested and the lipid-soluble pigments were extracted in equal volumes of 100% acetone. Extracts were initially analyzed spectrophotometrically between 330 nm to 730 nm, a strong absorption peak at 384 nm indicating that scytonemin had accumulated in the cells [(2)]. Following UVA exposure, water-soluble compounds were also extracted from whole cells in equal volumes of 25% aqueous methanol. 50 µl of concentrated acetone or methanol extracts from cells exposed to UVA radiation were also analyzed by high pressure liquid chromatography (HPLC on a Waters e2695 equipped with a Supelco Discovery HS F5-5 column connected to a Waters 2998 PDA UV-Vis diode array detector using previously described conditions [(20,13)] with the following revisions [(13)]: flow rate was 1.0 mL /min-1 with linear gradients of 10 mM ammonium formate buffer (pH 3 adjusted with hydrochloric acid) and acetonitrile, (0-1.5 minutes 0-25% acetonitrile, 1.5-7.5 minutes 25-100% acetonitrile, 7.5-12.5 minutes 100-100% acetonitrile, 12.5-27.5 minutes 100-25% acetonitrile). Carotenoids, chlorophyll a, and scytonemin were monitored in the chromatograms at 384 nm, but spectra were recorded continuously between 200-800 nm using the PDA detector. Individual compounds were identified by their characteristic absorption maxima and appropriate retention time.

To obtain an authentic standard of the scytonemin monomer (Compound 1 in Figure 1) the results of Malla and Sommer [13] were repeated by cloning genes *scyA*, *scyB*, and *scyC* into the *NcoI*/*SacI* site of the pRSF-Duet-1 vector to produce the pRSF-ScyABC recombinant plasmid. Genes *scyE* and *scyF* were then cloned into the *NdeI*/*FseI* site of pRSF-ScyABC to produce the pRSF-ScyABC-ScyEF recombinant plasmid. Recombinant strains were cultured, induced, and extracted as published [13].

Cellular characterization of mutant strains. Additional imaging was carried out using a Zeiss LSM800 laser scanning confocal microscope equipped with a Plan-Apochromat 63x 1.40 NA oil immersion objective with fluorescence excitation wavelengths as above. Further image processing for presentation purposes was done using Zen 2.3.

Cellular localization of ScyE. To confirm bioninformatic predictions of ScyE localization, UVA induced and uninduced wild-type *N. punctiforme* cultures were harvested and subjected to lysis by osmotic shock as described by Ross *et. al.* 2007[28] with the following revisions: 300 mM sucrose lysis buffer was used to preferentially lyse the periplasmic membrane, while increasingly efficient whole cell lysates were obtained using 400 and 500mM lysis buffers. Proteomic analyses of lysate preparations were then conducted as follows: In-gel trypsinization was accomplished by heating 40 μg of each protein extract to 95°C in reducing Laemmli sample buffer (BioRad) for 10 minutes prior to separation on 4-20% TGX gels (BioRad). Gels were stained with Bio-Safe Coomassie G-250 Stain (BioRad) as per manufacturer's instructions. Each sample lane on an SDS-PAGE gel was cut into six equal size slices and placed into polypropylene tubes prior to destaining twice in water and twice in 50% acetonitrile (ACN; Sigma). Proteins were then reduced in 10mM dithiothreitol (DTT; Promega), and alkylated with 2.5mM ioadacetamide (IAA, Sigma), prior to a 37°C overnight digestion with trypsin (Promega)

in 25mM ammonium bicarbonate (Sigma). The peptides were then extracted from the gel pieces using 5% formic acid (FA) in ACN, and dried down in a speed vacuum, and analyzed using LC-MS/MS. Briefly, each digest was loaded onto a Dionex UltiMate® 3000 RSLC liquid chromatography (LC) system (Thermo, San Jose, CA). Peptides were separated using a 500 nL/min LC gradient comprised of 2%-60% B in 0-120 min. Mobile phase A was 2% ACN in water with 0.1% FA and mobile phase B was ACN/methanol/water (80/10/10 by volume) with 0.1% FA. Eluting peptides were directly injected into an Orbitrap Elite mass spectrometer (Thermo-Fisher) using collision-induced dissociation (CID) in positive ion mode. The instrument was configured to operate by data-dependent mode by collecting MS1 data at 60,000 resolving power (measured at m/z 275) with an automatic gain control (ACG) value of $1*10^6$ over a m/z range of 275-1800. Precursors were fragmented with normalized collision energy (NCE) of 35, fragments measured at 17,500 resolving power and a fixed first mass of 275 Da. Resulting tandem MS/MS were collected on the top 20 precursor masses present in each MS1 using an AGC value of $1*10^5$, max ion fill time of 50ms, and an isolation window of 1.5 Da. Database searching was performed using Mascot (Matrix Science) in Proteome Discoverer v1.4.0.288 (Thermo) against the *Nostoc punctiforme* ATCC29133 proteome (Proteome ID: UP000001191, Feb. 4, 2017 release date). Searches were performed using a fragment tolerance of 0.80 Da (Monoisotopic), parent Tolerance of 10 ppm (Monoisotopic), with Carbamidomethyl as a fixed and Oxidation as variable modifications and a maximum missed cleavages allowed of 2. False discovery rate was 0.6%. Normalization of spectral counts and generation of normalized spectral abundance factors were performed in Scaffold (v4.8.4, Proteome Software Inc.).
