# Supplementary material for: The Widely Conserved ebo Cluster Is Involved in Precursor Transport to the Periplasm during Scytonemin Synthesis in Nostoc punctiforme
Source: mBio. 2018 Nov 27;9(6):e02266-18. doi: 10.1128/mBio.02266-18 (PMC6282210; doi:10.1128/mBio.02266-18)
Supplement: TABLE S2 [file mbo006184193st2.pdf]

Supplementary Table S2

ebo cDNA target	Forward primer	Reverse primer
eboB	GCTAGTCAGTTTGGCATCCAACAC	GCTTCTGTCATGTCGTCATAGCC
eboC	CCGCCGCCGTATAAACCACTTG	ATCGCGCTCGAGGCTTGTAGGTTTCG
eboE	CCATATTGATTTAGAACCTGAGCCTGATGG	ACGCGCAAACACAGATTGTGG
eboF	GGGTAAACAAGCCTACCATC	GGAAATTTTATTTGCGGATCGAGG
